# Genetic Basis of Variation in Rice Seed Storage Protein (Albumin, Globulin, Prolamin, and Glutelin) Content Revealed by Genome-Wide Association Analysis

**DOI:** 10.3389/fpls.2018.00612

**Published:** 2018-05-09

**Authors:** Pingli Chen, Zhikang Shen, Luchang Ming, Yibo Li, Wenhan Dan, Guangming Lou, Bo Peng, Bian Wu, Yanhua Li, Da Zhao, Guanjun Gao, Qinglu Zhang, Jinghua Xiao, Xianghua Li, Gongwei Wang, Yuqing He

**Affiliations:** ^1^National Key Laboratory of Crop Genetic Improvement and National Center of Plant Gene Research, Huazhong Agricultural University, Wuhan, China; ^2^Life Science and Technology Center, China National Seed Group Co., Ltd., Wuhan, China

**Keywords:** GWAS, storage protein, grain quality, endosperm, nutrition, *Oryza sativa* L.

## Abstract

Rice seed storage protein (SSP) is an important source of nutrition and energy. Understanding the genetic basis of SSP content and mining favorable alleles that control it will be helpful for breeding new improved cultivars. An association analysis for SSP content was performed to identify underlying genes using 527 diverse *Oryza sativa* accessions grown in two environments. We identified more than 107 associations for five different traits, including the contents of albumin (Alb), globulin (Glo), prolamin (Pro), glutelin (Glu), and total SSP (Total). A total of 28 associations were located at previously reported QTLs or intervals. A lead SNP sf0709447538, associated for Glu content in the *indica* subpopulation in 2015, was further validated in near isogenic lines NIL(Zhenshan97) and NIL(Delong208), and the Glu phenotype had significantly difference between two NILs. The association region could be target for map-based cloning of the candidate genes. There were 13 associations in regions close to grain-quality-related genes; five lead single nucleotide polymorphisms (SNPs) were located less than 20 kb upstream from grain-quality-related genes (*PG5a*, *Wx*, *AGPS2a*, *RP6*, and, *RM1*). Several starch-metabolism-related genes (*AGPS2a*, *OsACS6*, *PUL*, *GBSSII*, and *ISA2*) were also associated with SSP content. We identified favorable alleles of functional candidate genes, such as *RP6*, *RM1*, *Wx*, and other four candidate genes by haplotype analysis and expression pattern. Genotypes of *RP6* and *RM1* with higher Pro were not identified in *japonica* and exhibited much higher expression levels in *indica* group. The lead SNP sf0601764762, repeatedly detected for Alb content in 2 years in the whole association population, was located in the *Wx* locus that controls the synthesis of amylose. And Alb content was significantly and negatively correlated with amylose content and the level of 2.3 kb *Wx* pre-mRNA examined in this study. The associations or candidate genes identified would provide new insights into the genetic basis of SSP content that will help in developing rice cultivars with improved grain nutritional quality through marker-assisted breeding.

## Introduction

Rice is one of the major staple cereal foods and is an important source of total protein in human food. SSP account for approximately 8% of the dry grain weight and are the second most abundant ingredient after starch in rice. Rice has the lowest protein content among cereal grains, but net protein utilization is highest (Juliano, 1992). High-protein rice is likely to increase human nutrition in poor families, especially where rice is a staple food. Therefore, increasing the SSP content has become one of the main breeding objectives in improving nutritional quality in rice.

The SSP in rice can be classified into four fractions: albumin, globulin, prolamin, and glutelin, according to differences in solubility. Glutelin encoded by 15 genes accounts for as much as 80% of the total SSPs and is concentrated in the milled fraction, whereas prolamin, the most evenly distributed protein, accounts for less than 5% (Yamagata et al., 1982). Based on amino acid sequence similarity, glutelins are classified into four subfamilies: GluA, GluB, GluC, and GluD (Kawakatsu et al., 2008). In rice, SSP genes have been cloned and characterized mostly by mutant screening (Ren et al., 2014). Glutelins are synthesized in the rough endoplasmic reticulum as a 57 kDa precursor. Previous studies have identified rice 57H mutants that accumulate relatively high levels of 57 kDa pro-glutelin and have floury/opaque endosperm phenotype (Wang et al., 2009; Ren et al., 2014). Of 57H mutants, only *gpa3*, *Osvpe1*, and *OsRab5a* have been successfully cloned (Wang et al., 2009; Wang Y. et al., 2010; Ren et al., 2014). Prolamins are encoded by a multigene family of 34 gene copies. Based on the molecular mass, prolamins classified into three groups: 10 kDa prolamin (RP10), 13 kDa prolamin (RM1, RM2, RM4, and RM9), and 16 kDa prolamin (RP16) (Yamagata et al., 1982; Kawakatsu et al., 2008). Both albumin and globulin are concentrated in the bran and polishing during milling removes a major portion of these proteins (Shewry, 2007). Globulin is also easily digested (Yamagata et al., 1982; Zhang et al., 2008), and only a limited number of genes has been cloned and characterized (Bhullar and Gruissem, 2013). RA16 and RA17 that are associated with seed allergenic protein have been reported as albumin genes in previous studies (Adachi et al., 1993). The nutritional value of glutelin is higher than prolamin because it has a greater digestive capacity by humans and higher lysine content in rice (Ogawa et al., 1987). However, patients with kidney disease and diabetes need low glutelin diet (Mochizuki and Hara, 2000; Nishimura et al., 2005; Morita et al., 2009). Some proteins that belong to the albumin and globulin are considered to be allergenic. Collectively, emphasis in rice breeding should not only be on the concentration, but also on the quality of rice protein.

Seed storage protein is quantitatively inherited, and is affected by growing environment (Shewry, 2007). QTL mapping based on molecular markers and linkage maps has always been a common method of genetic studies (Li et al., 2014; Peng et al., 2014). Many QTL for crude protein content in rice have been reported (Aluko et al., 2004; Wang et al., 2008; Lou et al., 2009; Yu et al., 2009; Peng et al., 2014), but fewer studies have investigated the individual protein fractions in milled rice. *qPC1* (*OsAAP6*), controlling the natural variation in SSP content, has been cloned using a map-based cloning strategy (Peng et al., 2014). Zhang et al. (2008) identified 16 QTL for contents of crude protein and the four protein fractions.

Genome-wide association study (GWAS) by means of single nucleotide polymorphism (SNP) has become the method of choice for investigation of the genetics of important traits in *Arabidopsis thaliana* (Chan et al., 2011), rice (Huang et al., 2010), maize (Xiao et al., 2016), sorghum (Morris et al., 2013), and others (Ogura and Busch, 2015). Although GWAS is widely used in genetic analysis of grain quality traits in rice, such as gelatinization temperature, amylose content, grain appearance, and milling quality (Borba et al., 2010; Huang et al., 2010), few studies have used this approach to investigate total SSP in rice (Huang et al., 2012; Bryant et al., 2013). The analysis of genetic basis of nutritional quality has been reported in maize (Deng et al., 2017). Maize *opaque2* (*o2*) mutation could increase free lysine levels and create the foundation for quality protein maize (QPM) breeding. Combining GWAS and linkage mapping, a gene duplication at the 27-kDa γ-zein locus *qγ27* was identified (Liu et al., 2016). *qγ27* increases the level of 27-kDa γ-zein gene expression in QPM, which is essential for endosperm modification. GWAS on amino acids has been carried, and 247 and 281 significant loci were identified in two different environments (Deng et al., 2017). However, no studies have been reported about the genetic basis of the four SSP fractions by GWAS in rice.

In this study, we performed GWAS of Alb, Glo, Pro, Glu, and total SSP in milled rice using 527 *Oryza sativa* accessions grown in two environments with an aim to identify loci involved in the genetics. Haplotype analysis and expression pattern of candidate genes then provided valuable in better understanding the genetic basis of variation in SSP content. The results shown here promote our understanding of the genetic basis of the storage protein groups, should be of use for breeders attempting to improve nutritional quality by means of marker assisted selection.

## Materials and Methods

### Plant Materials, Field Experiments, and Trait Measurements

A diverse worldwide collection of 527 *O. sativa* landraces and elite accessions (Supplementary Table S1) was used in this study. Structural analysis indicated that the entire collection belonged to nine subpopulations: *indI*, *indII*, *indica* intermediate, *Tej*, *Trj*, *japonica* intermediate, *Aus*, *VI*, and intermediate (Chen et al., 2014) and are available at the RiceVarMap^1^. The *indica* subpopulation (*indI*, *indII*, and *indica* intermediate) included 294 accessions, whereas the *japonica* subpopulation (*Tej*, *Trj*, and *japonica* intermediate) included 155 accessions.

Lines were planted in two environments in Hubei province: Ezhou in 2014 (Env. 1) and Wuhan in 2015 (Env. 2). The sowing dates were 25 May in both years. Seedlings about 25 days old were transplanted to the field. There were three rows with 10 plants each in each plot. The planting density was 16.5 cm between plants within a row, and 26.4 cm between rows. Field management basically followed recommended practice of agriculture, with fertilizer applied (per hectare) as follows: 48.75 kg nitrogen, 58.5 kg phosphorous, and 93.75 kg potassium as the basal fertilizer; 86.25 kg nitrogen at the tilling stage; and 27.6 kg nitrogen at the booting stage.

At maturity, three plants in the middle of the second row of each accession were harvested and bulked. Dry seeds were threshed in bulk and the rough rice was air-dried, and stored at room temperature for 3 months, and then stored at 4°C. 50 g of rough rice were dehulled into brown rice using a TR 200 dehuller (Kett, Tokyo, Japan). The embryo and aleurone layer of brown rice were removed into milled rice through a Pearlest mill (Kett, Tokyo, Japan). The rice was ground into flour with a CT 410 Cyclotec mill (FOSS, Hillerod, Denmark), passed through an 80-mesh sieve and stored at -20°C until the Alb, Glo, Pro, and Glu contents were determined based on previously published previous methods (Kumamaru et al., 1988). Briefly, 0.1 g sample of milled rice flour was placed in a centrifugation tube with 1.0 ml solvent containing 10 mM Tris–HCl buffer (pH7.5) for Alb extraction; 1.0 ml solvent containing 1 M NaCl, for Glo extraction; 60% n-propanol containing 1 mM EDTA-2Na, for Pro extraction; and 0.05 M NaOH for Glu extraction. The mixture was stirred for 2 h at room temperature, and extracts were separated from residues by centrifugation at 12,000 rpm for 15 min at 4°C The procedure was repeated three times. The extracts were stored at -20°C until further analysis. The contents of each fraction were determined by the Coomassie brilliant blue G-250 dye-binding method (Bradford, 1976) using bovine serum albumin as a standard, and quantitative analysis was carried out using Infinite M200 (Tecan Group, Männedorf, Switzerland) (Peng et al., 2014). Total SSP was the sum of the Alb, Glo, Pro, and Glu contents. The 2-year field experiment was designed with three replicates per year. The average SSP contents across three replicates within 1 year were used for GWAS. The SSP contents of the 527 *O. sativa* accessions are listed in Supplementary Table S1. Amylose content was measured as previously described method (Tan et al., 1999).

### Genome-Wide Association Study

All 527 accessions were genotyped via sequencing (Chen et al., 2014). SNP information was available on RiceVarMap (see foot note text 1), a comprehensive database for rice genomics. The physical locations of the SNPs were identified based on the rice annotation version 6.1 of variety Nipponbare from Michigan State University. A total of 3,916,415 SNPs in the whole population; 2,767,159 SNPs in the *indica* subpopulation; and 1,857,845 SNPs in the *japonica* subpopulation (minor allele frequency ≥0.05; number of accessions with minor alleles ≥6) was used for GWAS (Chen et al., 2014). A linear mixed model (LMM) was used for detecting associations using Fast-LMM (Lippert et al., 2011). Population structure was controlled using a kinship matrix constructed with all SNPs (Chen et al., 2014). Effective independent SNPs were detected (Li et al., 2012), and were 757,578 in the whole population; 571,843 in the *indica* subpopulation; and 245,348 in the *japonica* subpopulation. The thresholds were set at a *P-*value of 5.0 × 10^-6^ to identify significant association signals. To obtain independent association signals, multiple SNPs, exceeding the threshold in a 5 Mb region, were clustered based on an *r*^2^ of LD ≥ 0.25, and SNPs with the minimum *P*-value in a cluster were deemed to be lead SNPs.

### Statistical Analysis

Based on the standardized disequilibrium coefficients (D’), linkage disequilibrium (LD) was investigated. LD heatmaps were constructed using the TASSEL5.0^2^ program and R package “LDheatmap”^3^. Statistical analysis, including a correlation analysis, was conducted using IBM SPSS Statistics 22.0. Differences in SSP values were examined by Student’s *t*-tests. Broad-sense heritability (*H^2^*) for each phenotype was estimated using repeatability between 2 years of phenotypic data, calculated as the variance among variety grand means divided by their total phenotypic variance.

### Candidate Genes and Haplotype Analysis

Candidate genes within a 200 kb genomic region ( ± 100 kb from the lead SNP) in the associated loci were selected based on (i) biochemically related proteins or protein clusters; (ii) homologous genes with known function, and (iii) expression profiles. The genotypes of *RP6*, *RM1*, *Wx*, *PROLM1*, and other three candidate genes in the 527 rice accessions were obtained from the RiceVarMap database (see foot note text 1). The haplotypes were classified according to all SNPs (except sites in intron) including their intragenic region and 2 kb upstream with an MAF *>* 0.05 in a candidate gene. There were at least five rice accessions in the haplotypes for comparative analysis. One-way ANOVA and Student’s *t*-tests were applied to compare differences in SSP content among all possible haplotype pairs.

### RNA Extraction and Quantitative RT-PCR Analysis

According to the manufacturer’s instructions, the total RNA was extracted from rice different tissues using TRIzol reagent (Invitrogen). About 3 μg of RNA sample was processed by RNase-free DNaseI (Invitrogen) and reverse transcribed using M-MLV reverse transcriptase (Invitrogen) with Oligo(dT)15. Quantitative RT-PCR was carried out using Fast Start Universal SYBR Green Master (Rox) superMIX (Roche, Mannheim, Germany) in a ViiA 7 Real-Time PCR system (Applied Biosystems), according to the manufacturer’s introductions. Measurements were obtained using the relative quantification method. *Actin* was used as a reference gene in the qRT-PCR experiments. The experiment was designed with three biological replicates and three technical replicates per material. Error bars indicate standard error. The measurements were obtained using the relative quantification method. The significant difference was analyzed statistically by One-way ANOVA and Student’s *t*-tests. All primers for qRT-PCR analysis are listed in Supplementary Table S2.

## Results

### Phenotypic Variation and Heritability of SSP Content

**FIGURE 1:**
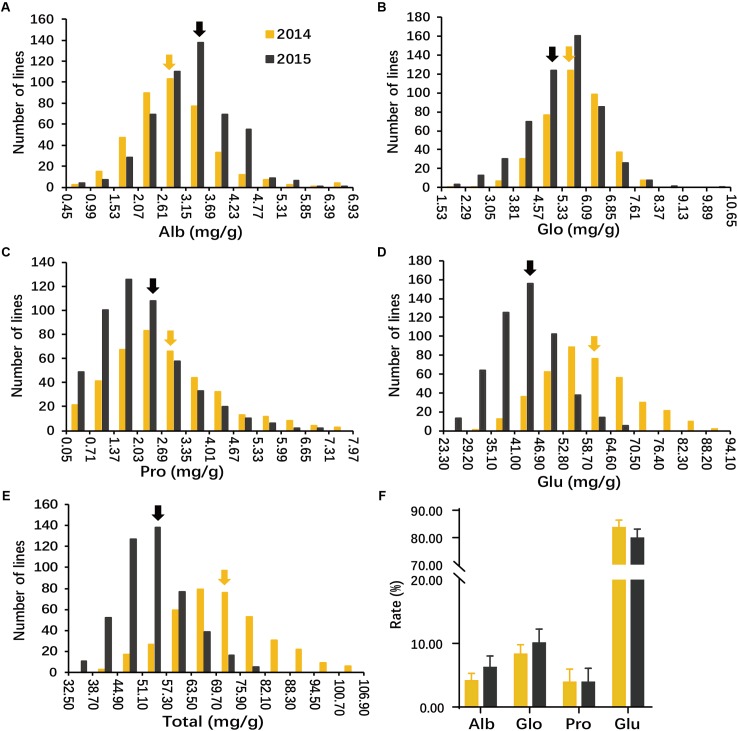
Phenotypic distribution of seed storage proteins (SSPs) of milled rice in the whole population. Histograms show the distributions of albumin (Alb) **(A)**, globulin (Glo) **(B)**, prolamin (Pro) **(C)**, glutelin (Glu) **(D),** and Total SSP **(E)** of milled rice measured in two environments (2014 and 2015). Arrowheads indicate mean values. **(F)** Proportions of individual components in total protein in 2014 and 2015 across all accessions. *y*-axis ‘Proportion’. Error bars, SE of replicates.

The result analysis revealed a large variation in all phenotypes evaluated and the traits appeared to be normally distributed (**Figures 1A–E** and Supplementary Table S3). Average SSP contents in Env. 1 and 2 were 70.9 and 53.7 mg/g; Alb was 2.9 and 3.3 mg/g; Glo was 5.8 and 5.3 mg/g; Pro was 2.8 and 2.1 mg/g; and Glu was 59.4 and 43.5 mg/g, respectively (**Figures 1A–E**). Glu accounted for approximately 80% of total SSP; Alb and Pro each accounted for about 5%; and Glo accounted for about 10% (**Figure 1F**). Compared with other three storage protein contents, the average content of Pro was the lowest, but the variation of Pro was largest in the whole population and each subpopulation in both environments. In four storage protein, Total SSP showed the lowest heritability (29.5%), whereas Pro showed the highest (76.8%) (Supplementary Table S3).

Correlation coefficients between each pair of components, and between components and total SSP were significant and positive in both environments, except for those between Glo and Pro in any environment and between Alb and Glo and between between Alb and Pro in Env. 2 (Supplementary Table S4). High correlations were found only between Glu and total SSP in both environments with coefficients of 0.99 in Env. 1 and 0.98 in Env. 2.

### Genome-Wide Association Study for SSP Contents

**FIGURE 2:**
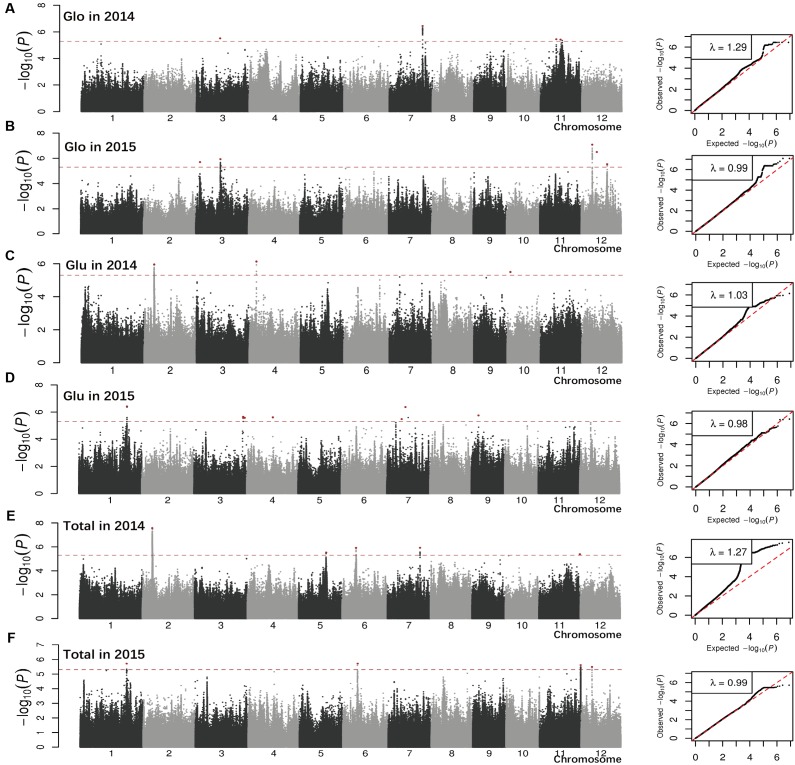
Genome-wide *P*-values and quantile-quantile plots from linear mixed model (LMM) model for Glo **(A,B)**, Glu **(C,D),** and Total SSP **(E,F)** in 2 years across all accessions. The *x-*axis depicts the physical locations of single nucleotide polymorphisms (SNPs) across the 12 rice chromosomes and the *y-*axis is the –log10 (*P-*value). Lead SNPs in significant peaks are red. The horizontal dotted line indicated the genome-wide significance threshold (*P* = 5.0E-06). Total, total SSP content.

We performed GWAS on the entire population and on the *indica* and *japonica* subpopulations for each year. The FaST-LMM program reduced the effect of population structure (Yang et al., 2014). Quantile-quantile plots of all five traits for the whole population, and *indica* and *japonica* subpopulations are illustrated in **Figure 2** and Supplementary Figures S1, S2. Some associations were detected in different subpopulations, and some of the associations for different traits were in the same chromosomal regions. Any two lead SNPs within a 100 kb region were considered to be a single association locus.

The association analysis for the whole population identified 34 loci (phenotypic variance >10%) associated with three traits with a suggestive threshold value at 5.0E-06 (**Table 1**). Most of them were detected for Alb (21 associations) and Pro (12 associations). Lead SNPs for Alb were widely distributed in the rice genome: chromosome 1, 2, 3, 4, 5, 6, 7, 8, and 9, with chromosomes 6 and 7 having more associations. Associations explained phenotype variation of 10.4–20.9%, with the association on chromosome 6 (sf0601764762) making the largest effect. For Pro, associations accounting for 10.1–34.6% of the phenotypic variance were identified on chromosomes 2, 5, 7, 10, and 11, with chromosome 5 and 7 exhibiting more associations. Only one lead SNP sf0317000156 on chromosome 3 with a phenotype variation of 14.4% was detected for Glo. No lead SNP with phenotype variation of more than 10% was detected for Glu or total SSP.

**Table 1:** Associated single nucleotide polymorphisms (SNPs) identified by linear mixed model (LMM) method in the whole population.

Trait	Chr.	Lead SNP^a^	Neighboring gene (kb)^b^	2014		2015		Chr interval/Previous QTL	Reference^c^
							
				*P*_LMM	P.V (%)	*P*_LMM	P.V (%)		
Alb	1	sf0103375439		3.7E-07	10.9				
Alb	2	sf0224727849		4.7E-07	11.2			2–12	Wang et al., 2008
Alb	3	sf0301966877		4.2E-08	10.7				
Alb	3	sf0303598854		7.7E-07	13.7				
Alb	3	sf0324084881		1.1E-06	12.4				
Alb	4	sf0413224092				2.6E-06	10.5		
Alb	5	sf0500466702				5.5E-07	11.3		
Alb	5	sf0505469121		1.2E-06	11.0				
Alb	5	sf0517961649				1.9E-06	11.3		
Alb	6	*sf0601635034*				2.5E-07	14.5		
Alb	6	sf0601764762	*Wx* (0.0)	2.0E-08	12.9	8.6E-16	20.9	6–2; *qPC-6*; *pro6*	Aluko et al., 2004; Wang et al., 2008; Lou et al., 2009; Yu et al., 2009
Alb	6	sf0601825636				8.5E-09	10.4		
Alb	6	sf0607130088		3.8E-10	17.4				
Alb	7	**sf0710433511**		2.1E-07	14.2				
Alb	7	sf0712003068		5.6E-07	13.4				
Alb	7	sf0719625273				3.7E-06	11.8		
Alb	7	sf0721422456		9.0E-07	11.7				
Alb	7	sf0724600666		5.8E-07	12.8				
Alb	8	sf0806546258		1.3E-06	13.0				
Alb	8	sf0815666771	*AGPS2a* (3.1)			1.3E-06	12.2	8–9; 8–10; 8–14	Wang et al., 2008
Alb	9	sf0915262039		6.2E-08	13.8				
Glo	3	sf0317000156				1.2E-06	14.4		
Pro	2	sf0202790542		1.2E-06	10.1				
Pro	5	sf0515030424				2.1E-12	13.2		
Pro	5	sf0515265287	*PG5a* (15.4); *Prol14* (75.8)	1.6E-06	6.0				
Pro	5	**sf0514987630**		5.6E-09	18.7	2.1E-12	13.2		
Pro	5	sf0515211855		6.5E-07	8.5	2.7E-11	13.7		
Pro	5	**sf0519612378**	*Flo4* (43.6)	1.2E-16	25.8	5.9E-11	14.9		
Pro	7	**sf0705739605**	*RM1* (1.2); *RP6* (-2.7)	6.5E-11	24.7	5.4E-16	26.3	7–4; 7–5	Wang et al., 2008
Pro	7	**sf0705159012**		1.1E-06	20.4				
Pro	7	**sf0706210463**	*RA17* (44.2); *RA16* (65.4)	1.30E-13	34.6	1.2E-13	23.0		
Pro	7	**sf0705534834**				4.7E-12	11.3		
Pro	7	**sf0706363663**		1.3E-17	33.1	1.2E-13	23.0		
Pro	10	sf1002878232		4.6E-07	18.8			10–1	Wang et al., 2008
Pro	11	sf1119231723				1.5E-06	16.0		
Glu	3	sf0335020536	*GPA3* (87.6)			2.6E-06	2.5		
Glu	7	sf0712842943	*GBSSII* (72.3)			4.3E-07	4.3	7–4; 7–5	Wang et al., 2008
Total	1	sf0132041148	*GluA1* (34.5); *OsAAT2* (-38.5)			1.9E-06	7.0	*Pro1*	Aluko et al., 2004
Total	5	sf0519017245	*ISA2* (75.2)	3.0E-06	7.2				


*Pop, population; Chr., chromosome; P, P-value estimated in LMM; P.V (%), proportion of phenotypic variance explained; Total, total seed storage protein (SSP) content. ^*a*^Associated SNPs within 100 kb for the same trait are considered the same locus; for Alb, Glo, and Pro, only lead SNPs with P.V (%) more than 10% are shown; bold scripts indicate detections in different populations and italic indicates detection in different traits.^*b*^Negative value means the gene is upstream of the SNP site.^*c*^Refers to interval or QTL reference.*

A large number of peaks (phenotypic variance >10%) were also detected by GWAS in the *indica* (33 associations) and *japonica* (40 associations) subpopulations (Supplementary Table S5). For Alb, 23 associations were distributed on all 12 rice chromosomes, except chromosome 3 and 10, but only three associations were identified in the *indica* subpopulation. Alb associations identified in the *indica* and *japonica* subpopulations explained phenotype variation of 10.5–12.3, and 11.3–41.6%, respectively. Four SNPs, sf0142207782, sf0209990680, sf0605251091, and sf0804866973 individually explaining more than 30% of the Alb variation, were detected in the *japonica* subpopulation in Env. 1. For Glo, 14 associations were identified on chromosomes 1, 3, 4, 5, 6, 9, 11, and 12, and equal numbers of associations were detected in the *indica* and *japonica* subpopulations, explaining 10.3–16.8 and 12.9–17.0% of the variation, respectively. For Pro, there were 23 associations, involving chromosomes 1, 3, 4, 5, 7, 9, 11, and 12. Among them 14 (with phenotype variation of 10.4–38.3%) and 9 (with phenotype variation of 15.5–22.6%) associations were identified in the *indica* and *japonica* subpopulations, respectively. Ten associations for Glu were detected on chromosomes 1, 4, 5, 6, 7, 8, 9, and 10, in both subpopulations. For total SSP, only three associations on chromosomes 10 and 11 with phenotype variation of 10.4–14.8% were detected in the *indica* subpopulation.

Among the 107 associations detected in the whole population and in the *indica* and *japonica* subpopulations, 16 were detected in different populations and nine involved two or three different traits (**Table 1** and Supplementary Table S5). Examples include lead SNP sf0519612378 with phenotypic variance of 14.9–36.6% that was detected in the whole population and *indica* subpopulation in Env. 1 and 2; and lead SNP sf1022972496 detected in *indica* subpopulation in Env. 1 was associated with phenotypic variances of 12.0 and 14.8% for traits Glu and total SSP, respectively. Seven associations in the whole population and four associations in the *indica* subpopulation were detected both in both environments. For Pro, two lead SNPs (sf0514987630 and sf0515211855) in the whole population and one lead SNP (sf0515706446) in the *indica* subpopulation were detected in both environments. The significance levels of the associations ranged from *P* = 5.0E-06 to *P* = 8.6E-16, *P* = 4.9E-06 to *P* = 9.0E-08, *P* = 5.0E-06 to *P* = 1.3E-17, *P* = 2.1E-06 to *P* = 1.4E-08, and *P* = 4.5E-06 to *P* = 3.1E-08 in LMM for Alb, Glo, Pro, Glu, and total SSP, respectively, and the most significant association for sf0706363663 located in chromosome 7 (**Table 1**).

### Co-localization of Associated Sites With QTLs Previously Reported and Grain Quality-Related Genes

There were many overlaps between the present associations detected by GWAS and reported QTLs or intervals related to SSP content in rice. A total of 28 associations from this study were located at previously reported QTLs or intervals (shown in **Table 1** and Supplementary Table S5 with corresponding references) of which 10 and eight associations were for Alb and Pro, respectively.

**FIGURE 3:**
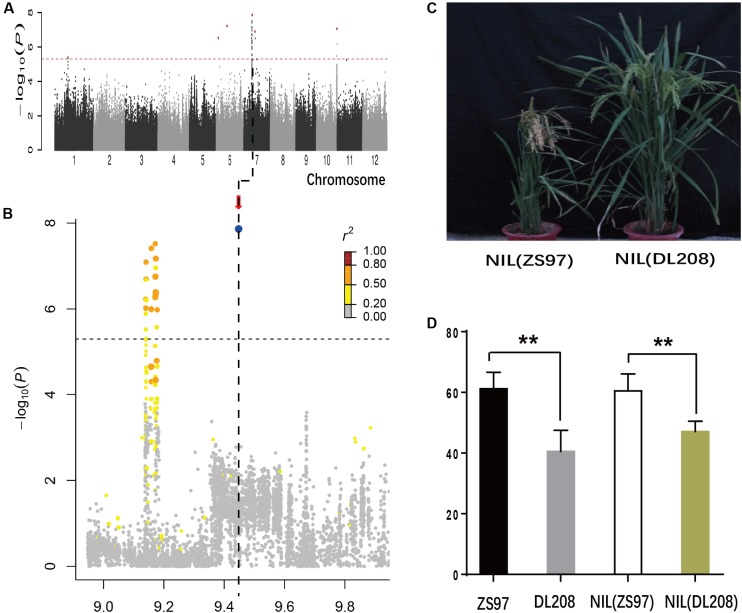
Genome-wide association study (GWAS) for Glu in 2015 and Validation of GWAS Signals by genetic materials for the chromosome 7 peak. Manhattan plots of LMM for Pro in the all accessions in 2015 **(A)**. **(B)** Local Manhattan plot surrounding the peak in 2015 on chromosome 7. Arrow indicates the position of the lead peak. The corresponding colors of *r^2^* represent linkage disequilibrium levels. **(C)** Plant architectures of near-isogenic lines. **(D)** Phenotypes of Glu of two parents ZS97, DL208, NIL(ZS97), and NIL(DL208). ^∗∗^Indicates the differences of Glu between two materials are significant at *P* < 0.01.

In the *indica* subpopulation, lead SNP sf0709447538 in a notable hotspot region at the interval of 9.1–9.5 Mb, explaining 12.7% of the Glu variation, was detected on chromosomes 7 in 2015 (**Figures 3A,B** and Supplementary Table S5). Interestingly, we found that the lead SNP sf0709447538 was overlapped with the amino acid content QTLs (7–4, 7–5, and 7–6), identified in a previous study using an F_9_ recombinant inbred line population, which derived from a cross between Zhenshan97 (ZS97) and Delong208 (DL208) (Wang et al., 2008; Zhong et al., 2011). To validate the QTL, we developed near-isogenic lines (NILs) (**Figure 3C**). NILs of QTL were developed by successive crossing and backcrossing ZS97 (high protein content) and DL208 (low protein content), three times (BC_3_) to ZS97. The QTL was selected by two molecular markers MRG186 and MRG4499 (Supplementary Table S2). Self-pollinating the BC_3_F_1_ plants heterozygous for this fragment produced NIL(ZS97) and NIL(DL208). Analysis of NIL(ZS97) and NIL(DL208) showed that NIL(ZS97) was significantly higher Glu than NIL(DL208), which was the same as the phenotype in two parents (**Figure 3D**). The result indicated that the QTL was reliable, which helps further identify the underlying genes and their genetic basis.

On the other hand, 13 associations were detected in regions close to previously identified grain-quality-related genes. Five genes (*PG5a*, *Wx*, *RM1*, *RP6*, and *AGPS2a*) were less than 20 kb from lead SNPs. *PG5a*, *RM1*, and *RP6* were associated for Pro; *Wx* and *AGPS2a* was associated with Alb. Glutelin genes *GluA1* and *OsAAT2* were associated with total SSP in Env. 2. Additionally, several lead SNPs were located close to starch-metabolism-related genes, such as *PUL*, *ISA2*, *Wx*, *GBSSII*, *OsACS6*, and *AGPS2a*. SNPs located close to other reported grain quality-related genes (*Prol14*, *RA17*, *RA16*, and *PGL*) were also examined using a LMM (**Table 1** and Supplementary Table S5).

### Haplotype Analyses for the Reported Genes *RP6* and *RM1*

**FIGURE 4:**
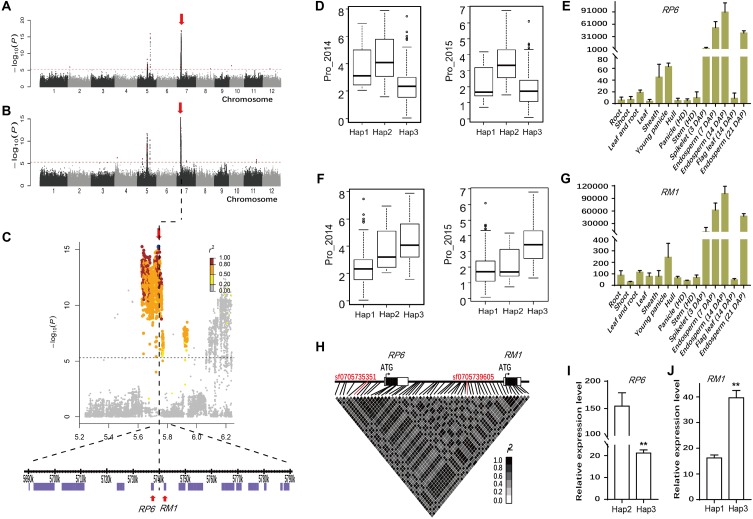
Genome-wide association study for Pro in two environments and identification of the causal gene for the chromosome 7 peak. Manhattan plots of LMM for Pro in the all accessions in 2014 **(A)** and in 2015 **(B)**. Arrowheads indicate the positions of strong peaks investigated in this study. **(C)** Local Manhattan plot surrounding the peak in 2015 on chromosome 7. Arrow indicates the position of the lead peak. The corresponding colors of *r^2^* represent linkage disequilibrium levels. The panel shows a 50 kb region on each side of the peak SNP, with annotated genes indicated by purple boxes. Previously identified genes (*RP6* and *RM1*) controlling prolamin content are labeled. The distribution of Pro values in 2014 (Left) and 2015 (Right) in the three haplotypes of *RP6* **(D)** and *RM1* **(F)**. Expression signals of *RP6* **(E)** and *RM1* **(G)** in various tissues of ZS97 based on the microarray data. The *y*-axis represents the expression signals. **(H)** Representation of pairwise *r*^2^ values among polymorphic sites in *RP6* and *RM1*. The lines in red represent lead SNP. Expression levels of *RP6* **(I)** and *RM1* **(J)** in the endosperm at 7 days after pollination in *indica* group. Error bars, SE of 3 replicates. ^∗∗^Indicates the differences of expression levels between two haplotypes are significant at *P* < 0.01. Hap, haplotype; HD, heading date; DAP, day after pollination.

The association SNP sf0705739605 for Pro was each <10 kb away from two prolamin genes *RP6* and *RM1* reported to encode prolamins in rice (Wen et al., 1993; Kawakatsu et al., 2009). In particular, the lead SNP with highly significant *P*-values (*P* = 6.5E-11 in Env. 1 and *P* = 5.4E-16 in Env. 2) was identified in the whole population and *indica* subpopulation (**Figures 4A–C**, **Table 1**, and Supplementary Table S5). The association explained 24.7 and 26.3% of the phenotypic variances in the whole population in Env. 1 and 2, respectively. Lead SNPs sf0705735351 and sf0705739605 and all polymorphic sites in *RP6* and *RM1* were in high linkage disequilibrium (in high LD with each other; *r*^2^ = 0.94–0.99) with most polymorphic sites (**Figure 4H**). We performed haplotype analyses for *RP6* and *RM1* and identified three main haplotypes (Hap1-3) at each locus (Supplementary Table S6). Hap1 and hap2 of *RP6* and hap2 and hap3 of *RM1* were not identified in *japonica*. Hap2 of *RP6* and hap3 of *RM1* had significantly higher Pro than the alternative haplotypes in both environments (**Figures 4D,F**). In the region contained coding region and 2 kb upstream of *RP6* and *RM1*, 17 and 26 SNPs were found, respectively (Supplementary Table S6). At the *RP6* locus, there were two synonymous SNPs, two non-synonymous SNPs in the exon, three SNPs in the 3′ untranslated regions, and 10 substitutions in the 2 kb *cis*-regulatory region. At the *RM1* locus, there were five synonymous SNP, no non-synonymous SNPs in the exon, two SNPs in the 5′ and 3′ untranslated regions, and 19 substitutions in the 2 kb *cis*-regulatory region. To get an overview of the expression profiles of *RP6* and *RM1*, the CREP database^4^, a website that contains the dynamic gene expression profile of *indica* rice, was searched (Wang L. et al., 2010). The results showed that *RP6* and *RM1* displayed high-level expressions in endosperm but low-level expressions in other tissues in ZS97 (**Figures 4E,G**). Considering the complexity of population structure and genetic background, we checked the expressions of *RP6* and *RM1* with different haplotypes in 20 and 34 accessions randomly chosen from *indica* group, respectively. Using qRT-PCR analysis, The results indicated that in the endosperm at 7 days after pollination (d.a.p.), expression levels of *RP6* in hap2 accessions were much higher than those in hap3 accessions (**Figure 4I** and Supplementary Table S7), and expression levels of *RM1* in hap3 accessions were much higher than those in hap1 accessions (*P* < 0.01) (**Figure 4J** and Supplementary Table S7). These results show that two genes might be good candidates for the GWAS locus. In conclusion, two genes had high-level and specific expressions in endosperm of rice, and genotypes with higher Pro exhibited much higher expression levels in *indica* group in this study.

### Analysis of One Candidate Gene *Wx*

**FIGURE 5:**
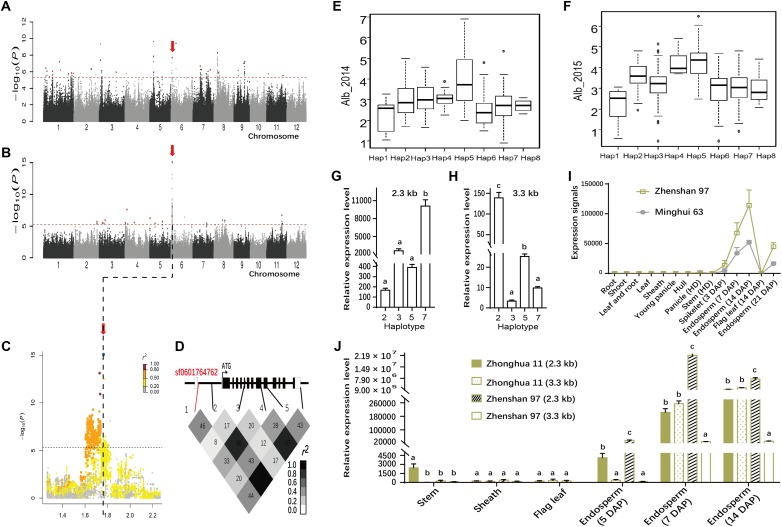
Genome-wide association study for Alb in two environments and identification of the causal gene for the peak on chromosome 6. Manhattan plots of LMM for Alb for all accessions in 2014 **(A)** and 2015 **(B)**. Arrows indicate the position of the lead peak. **(C)** Local Manhattan plot surrounding the peak in 2015 on chromosome 6. **(D)** Schematic of *Wx* structure (Top) and linkage disequilibrium (measured as pairwise *r*^2^ values) between the lead SNP sf0601764762 and some polymorphic sites in *Wx* (Bottom). The distribution of Alb values in 2014 **(E)** and 2015 **(F)** in the eight haplotypes of *Wx*. Quantity of 2.3 kb **(G)** and 3.3 kb **(H)** *Wx* in *indica* and *japonica* groups among four haplotypes in the endosperm at 7 days after pollination. **(I)** Quantity of 2.3 kb *Wx* RNA in various tissues of ZS97 and Minghui 63 based on the public microarray data. The *y*-axis represents the expression signals. **(J)** Comparison of the quantity of 2.3 and 3.3 kb *Wx* RNA between Zhonghua 11 and ZS97 with different genotypes by Real-time PCR. Error bars, SE of three replicates. Letters above the bars are ranked by Duncan test at *P* < 0.05; different letters next to SE bars indicate significant difference. Hap, haplotype; HD, heading date; DAP, day after pollination.

We also found a highly significant association signal for Alb involving sf0601764762 (*P* = 2.0E-08 in Env. 1 and *P* = 8.6E-16 in Env. 2) on chromosome 6 (**Table 1** and **Figures 5A–C**). The lead SNP, explaining 12.9 and 20.9% of the phenotypic variances in the whole population in Env. 1 and 2, respectively, was located in the first intron of *Wx* (**Table 1** and **Figure 5D**). Other SNPs in *Wx* showed different LD associations with sf0601764762 (**Figure 5D**). *Wx* is the most important genetic determinant of amylose content (Tian et al., 2009). We identified eight major *Wx* haplotypes with 23 SNPs (**Figures 5E,F** and Supplementary Table S6). Hap1, 6, and 7 showed lower Alb values than hap4 and 5 in both environments. The lead SNP sf061764762 is located to the first intron of *Wx*, the major gene determining starch content. This SNP generates alleles *Wx^a^* with a normal GT sequence at the 5′ splice junction of intron 1, and *Wx^b^* with a G to T mutation in intron 1. *Wx^a^* and *Wx^b^* produce a mature 2.3 kb *Wx* mRNA and a 3.3 kb *Wx* pre-mRNA, respectively (Wang et al., 1995). We checked the quantity of 2.3 and 3.3 kb *Wx* RNA with four haplotypes in 35 and 37 accessions randomly chosen from *indica* and *japonica*, respectively. Using qRT-PCR analysis, it showed that quantity of 2.3 kb *Wx* RNA in the endosperm at 7 d.a.p. in hap7 *indica* accessions were much higher than those in hap2 *indica* accessions, hap3 *japonica* accessions and hap5 *japonica* accessions (*P* < 0.01). In contrast, quantity of 3.3 kb *Wx* RNA in hap7 *indica* accessions were much lower than those in hap2 *indica* accessions and in hap5 *japonica* accessions (**Figures 5G,H** and Supplementary Table S7). With highest Alb content among four haplotypes, hap5 had relatively high 3.3 kb *Wx* RNA quantity and low 2.3 kb *Wx* RNA quantity. However, with lowest Alb content among four haplotypes, hap7 had relatively low 3.3 kb *Wx* RNA quantity and highest 2.3 kb *Wx* RNA quantity. It was found that *Wx* exhibited higher level quantity of 2.3 kb *Wx* mRNA in endosperm in ZS97 (hap7) than Minghui 63 (hap2), two *indica* cultivars (**Figure 5I**). We further compared the quantity of 2.3 and 3.3 kb *Wx* RNA in various tissues between two varieties Zhonghua 11 (hap2) and ZS97 by quantitative RT-PCR (**Figure 5J**). The results showed that ZS97 had lower 3.3 kb *Wx* RNA quantity and higher 2.3 kb *Wx* RNA quantity than Zhonghua 11 in endosperm of 7 and 14 d.a.p. However, *Wx* displayed very low-level expressions in stem, sheath, and flag leaf in both ZS97 and Zhonghua 11. Then we measured amylose content of the corresponding accessions in 2015 (Supplementary Table S7). The correlation analysis among Alb, amylose content, quantity of 2.3 and 3.3 kb *Wx* RNA was performed, and the results are presented in **Table 2**. Amylose content had significant correlations with both quantity of 2.3 and 3.3 kb *Wx* RNA, but amylose content was positively correlated with quantity of 2.3 kb *Wx* RNA and was negatively correlated with quantity of 3.3 kb *Wx* RNA, which was consistent with previous study (Wang et al., 1995). Significant and negative correlations were observed between Alb and amylose content or quantity of 2.3 kb *Wx* RNA (**Table 2**), suggesting that *Wx* may negatively regulate Alb. However, Alb and quantity of 3.3 kb *Wx* RNA had no significant correlations. We speculated that *Wx* might influence Alb content.

**Table 2:** Correlation coefficients of Albumin (Alb), AC, and expression levels.

	Alb	AC	2.3 kb	3.3 kb
Alb	1.00			
AC	-0.49^∗∗^	1.00		
2.3 kb	-0.41^∗^	0.43^∗^	1.00	
3.3 kb	0.14	-0.37^∗^	-0.36	1.00


**Alb of 46 accessions in 2015 was measured; AC, amylose content of 44 accessions in 2015; 2.3 kb, relative expression level of 2.3 kb Wx; 3.3 kb, relative expression level of 3.3 kb Wx. ^∗^P* < *0.05 and ^∗∗^P* < *0.01*.*

### Analyses of Four Candidate Genes

Association analysis at the haplotype level greatly increases mapping power (Han et al., 2016). We re-detected these grain-quality-related genes mentioned above at the haplotype level (Supplementary Table S8). Four SSP loci (*OsAAT2*, *RA17, RM1*, and *RP6*) and four starch-metabolism-related genes (*AGPS2a*, *ISA2, PUL*, and *Wx*) were detected in both environments; however, *GBSSII*, *GluA1*, and *RA16* were only detected in one environment. The order of magnitudes of the *P*-values for these genes was greatly reduced compared with that at the SNP level. To further verify the association possibility, we validated some of candidate genes detected via GWAS by haplotype analyses and expression profiles in public databases.

**FIGURE 6:**
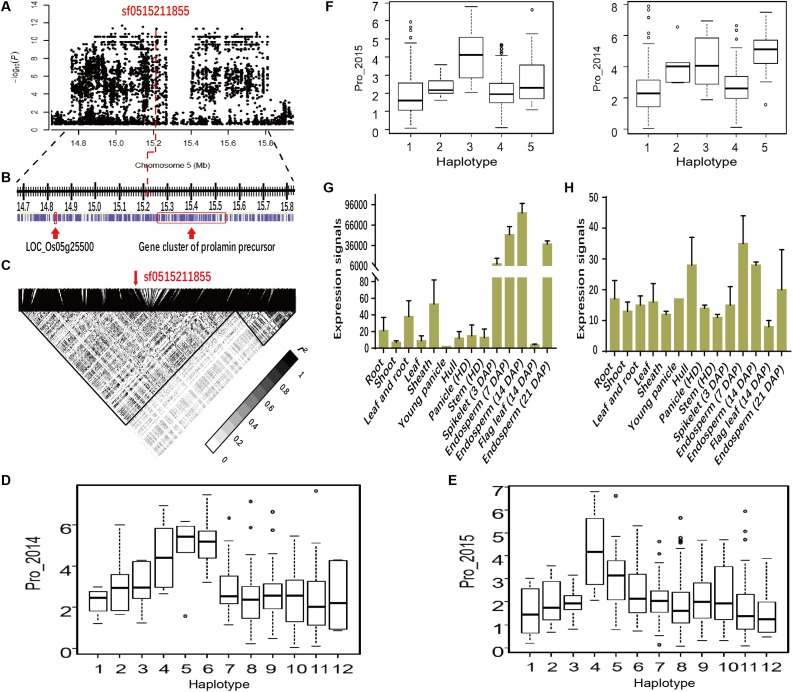
Regions of the genome showing association signals and the expression profiles of candidate genes. **(A)** An associated locus from MLM for Pro in 2015 in 14.6–16.0 Mb on chromosome 5. Arrow indicates the position of the lead SNP sf0515211855. **(B)** The panel shows a region with the candidate genes indicated by arrows. **(C)** LD heatmap surrounding the peak between 14.6–16.0 Mb on chromosome 5. The distribution of Pro values in 2014 **(D)** and 2015 **(E)** in the twelve haplotypes of the candidate gene (*PROLM1*). **(F)** The distribution of Pro values in 2014 (Left) and 2015 (Right) in the five haplotypes of the candidate gene (LOC_Os05g25500). Expression signals of *PROLM1* **(G)** and LOC_Os05g25500 **(H)** in various tissues of ZS97 based on the public microarray data. The *y*-axis represents the expression signals. HD, heading date; DAP, day after pollination.

**FIGURE 7:**
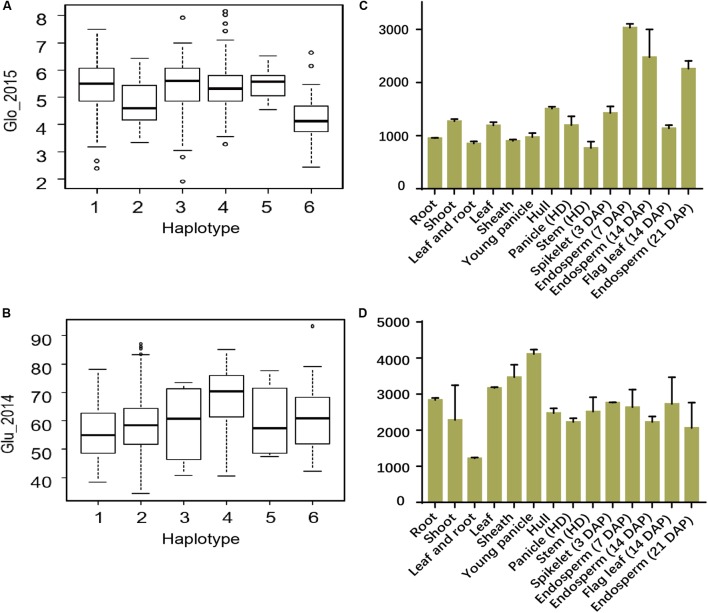
Haplotype analyses and expression profiles of candidate genes. The distribution of Glo values in 2015 in *indica* group in the six haplotypes of the candidate gene (LOC_Os03g29750) **(A)** and Glu values in 2014 in all group in the six haplotypes of the candidate gene (LOC_Os02g13130) **(B)**. Expression signals of LOC_Os03g29750 **(C)** and LOC_Os02g13130 **(D)** in various tissues of ZS97 based on the public microarray data. The *y*-axis represents the expression signals. HD, heading date; DAP, day after pollination.

To determine whether novel functional loci were implicated by GWAS, we investigated SNP sf0515211855 (*P* = 6.5E-07 in Env. 1 and *P* = 2.7E-11 in Env. 2), in Chr.5 (14.7-15.8 Mb) (**Figures 6A–C**). This lead SNP was significantly associated with Pro and explained 8.5 and 13.7% of the phenotypic variance in the whole population in Env. 1 and 2, respectively (**Table 1**). SNPs in the region were grouped into two LD block, and sf0515211855 was in the first block. Within this block, there were a gene cluster of 18 prolamin precursors and a candidate gene encoding an expressed protein (LOC_Os05g25500) (**Figure 6B**). Within the cluster we focused on *PROLM1* (LOC_Os05g26240), located close to the lead SNP, and identified twelve major haplotypes (Hap1-12) (**Figures 6D,E** and Supplementary Table S6). Hap4, 5, and 6 had significantly higher Pro than other haplotypes in both environments. Microarray data (see foot note text 4) indicated that *PROLM1* had extremely high expression enrichment in endosperm of ZS97 at the ripening stage (**Figure 6G**). We obtained five major haplotypes (Hap1-5) for LOC_Os05g25500 (Supplementary Table S6). Hap3 and 5 had significantly higher Pro content than Hap1 and 4 in both environments (**Figure 6F**). LOC_Os05g25500 exhibited higher expression levels in endosperm than other tissues of ZS97 (**Figure 6H**). We focused on other two candidate genes LOC_Os03g29750 (encoding an expressed protein) and LOC_Os02g13130 (encoding a KH domain-containing protein) for Glo in *indica* group in 2015 (**Figure 7A**) and Glu in all group in 2014 (**Figure 7B**), respectively. Hap5 of LOC_Os03g29750 had significantly higher Glo than other haplotypes, and LOC_Os03g29750 showed very high-level expression in endosperm of ZS97 (**Figure 7C**). Hap4 of LOC_Os02g13130 had significantly higher phenotypes than other haplotypes. For LOC_Os02g13130, homologous with maize gene encoding high molecular weight glutenin subunit x, it had high-level expressions in most tissues and organs (**Figure 7D**). These results indicated that these genes might be good candidates for the GWAS locus.

## Discussion

### Phenotypic Variation and Trait Correlation

The contents of the four components of SSP and the total SSP were normally distributed in two environments and influenced by environment in this study (**Figure 1**). The *indica* subpopulation showed wider variation in Pro than the *japonica* subpopulation, but narrower variation compared to *japonica* for Alb. These results indicated that there were probably different genetic bases underlying Alb and Pro in the two subpopulations. The contents and proportions of different components as well as total SSP in the present study were in agreement with those reported by Kumamaru et al. (1988). Glu is highest composition of SSP in rice endosperm and has more essential amino acid required for human (Tanaka et al., 1995), suggested that it is more effectively to improve protein content and nutritional value of rice by increasing Glu than other three SSPs.

In previous studies, different conclusions were reached on the heritability of SSP content in rice. Hillerislambers et al. (1973) reported that the heritability of SSP content was 13.3–37.2% in rice. However, Shenoy et al. (1991) obtained values as high as 71% and considered that it is effective to select protein content phenotypes in early generation in the protein content breeding. Here, different SSP showed different heritabilities (29.5–76.8%) (Supplementary Table S3). It suggested that four SSP may have differences in generation selection. To some extent, Alb with higher heritability was effectively selected in early generation, but it may not be good for Glu and Total with lower heritability. The relationship between different SSP components in rice is complex. In the present study, there were significant positive correlations between Glu and the other three SSPs in both environments (Supplementary Table S4), that was consistent with reported study (Zhang et al., 2008). These results indicated that Glu and other three SSPs might partially share the common genetic mechanism.

### GWAS for Seed Storage Protein in Rice

The use of high-density, genome-wide SNPs in GWAS not only can detect candidate genes, but also have a comprehensive understanding of the regulatory mechanism of related traits. Among the 107 associations detected in the study, few sites were repeatedly detected in both environments. Compared with Glo and Glu, Alb and Pro have larger variations in the populations (Supplementary Table S5). Similarly, association mapping was more efficient in identifying associations for Alb and Pro with relatively higher heritability than total SSP and Glu with lower heritability (Supplementary Tables S3–S5). On the other hand, we noticed that all associations detected for Alb and Pro explained more than 100% of the phenotypic variance. This suggests that phenotypic variance explained by the interaction between some of these associations might be very large.

Although genome-wide association studies are becoming more sophisticated, it should be noted that association mapping may lead to false positive associations largely caused by population structure. In order to reduce false-positives resulting from genetic structure, we also analyzed the *indica* and *japonica* panels separately by LMM. Thirteen associations were adjacent to previously known grain-quality-related genes (**Table 1** and Supplementary Table S5). These results suggested that association mapping was an effective way to find candidate genes for SSPs in rice. And 28 associations were detected in previously reported intervals or QTLs (Aluko et al., 2004; Wang et al., 2008). QTL *pro6* was repeatedly detected for Alb (sf0605251091 and sf0601764762) and Glu (sf0602321094). Lead SNPs (sf0709447538 and sf0712842943 for Glu, sf0705739605 and sf0706196757 for Pro) were co-located in intervals 7–4 and 7–5. Although large numbers of QTLs for grain protein content were detected in the past; only one major QTL has been cloned (Peng et al., 2014). For the lead SNP sf0709447538, explaining 12.7% of the Glu variation and located to the same chromosome region as QTL (7–4 and 7–5), we developed NILs to confirm the QTL. The results showed that it was significantly difference in Glu between two NILs and suggested that the QTL was reliable. To further purify the genetic backgrounds and fine map the QTL, it is needed to backcross the NILs for recombinant screening. It has been reported that a quantitative trait locus (*qγ27*) affecting expression of 27-kDa γ-zein has been successfully cloned by GWAS and linkage mapping analysis (Liu et al., 2016).

We detected a lead SNP responsible for Alb in the *Wx* gene region (**Figure 5** and **Table 1**), This region is considered a hotspot of major QTLs (6-2; *qPC-6*; and *pro6*) for protein content in rice (Aluko et al., 2004; Wang et al., 2008; Lou et al., 2009; Yu et al., 2009). Analysis of the correlation among Alb, amylose content and quantity of 2.3 kb *Wx* RNA showed that *Wx* might influence Alb content, that needs transgenic experiment to verify the function of *Wx*. Additionally, Starch-metabolism-related genes, such as *PUL* (Fujita et al., 2009), *ISA2* (Utsumi et al., 2011), *GBSSII* (Hirose and Terao, 2004), *Flo4* (Kang et al., 2005), and *AGPS2a* (Akihiro et al., 2005) were associated with SSPs in different populations. Overexpression of albumin gene *RAG2* significantly increased total protein content, prolamin, glutelin, and amylose content, but decreased total starch (Zhou et al., 2016). Carbon and nitrogen metabolisms show a cooperative modification, and consequently, validation the function of these associations might help to better understand the genetic relationship between SSP and starch contents in rice grain.

### Application in the Improvement of Grain Quality in Rice

Grain with good quality could be developed by regulating the SSP content. SSP is a typical quantitative trait typically affected by environment (Shewry, 2007). Combination of conventional breeding and molecular techniques, e.g., marker-assisted selection (MAS), may provide a more efficient approach for improving the SSP content of the grain than classical breeding alone (Zhang et al., 2008). The *Lgc1* mutation has been used to development new low easy-to-digest protein rice varieties with low glutelin content and high prolamin content, which is useful for patients with chronic renal failure (Mochizuki and Hara, 2000; Nishimura et al., 2005; Morita et al., 2009). *O2* mutation could increase free lysine and tryptophan levels by reducing the synthesis of zeins in maize, which is useful for QPM breeding (Mertz et al., 1964; Liu et al., 2016). Genes and possible causative SNPs identified in the present study could be used as potential targets for rice grain nutritional quality improvement. Glutelin is the most easily to digest and contains high lysine (Tanaka et al., 1995). The lead SNP sf0709447538 co-located in QTLs (7–4, 7–5, and 7–6) was validate to have an effect on glutelin, which could be targets for map-based cloning of the candidate genes to illuminate the molecular mechanism of glutelin and improve grain nutritional quality by MAS. *RM1* and *RP6* are adjacent genes on chromosome 7, and with higher levels expression and higher Pro, hap2 of *RP6* and hap3 of *RM1* are distributed mainly in the *indica* subpopulations (**Figure 4** and Supplementary Table S6). Therefore, the region covering *RM1* and *RP6* from *japonica* subpopulation can be a promising target for reducing Pro content and achieving better nutritional quality in *indica* cultivars. *Wx* may be a key gene for regulating the content of Alb and amylose content. The availability of the *Wx* gene sequence provides the possibility of improving the protein content via *Wx* gene modification. Different haplotype combinations of candidate genes for SSP would produce grains with different eating and nutritional quality. Genes and possible causative SNPs identified in the present study could be useful for breeding rice cultivars with favorable eating and nutritional quality.

## Conclusion

In the present study many associations were identified for five SSP traits using GWAS in two environments. We detected novel associations, known SSP genes, and SNPs adjacent to known starch-metabolism-related genes. We also analyzed haplotypes of known grain-quality-related genes. Our results suggested that GWAS was an effective way to identify genes for rice SSP traits and the level of 3.3 kb *Wx* pre-mRNA is positively correlated with Alb content, providing new insights into the genetic basis of rice quality. Overall, we provided useful information that could be used in future gene functional studies and rice quality improvement.

## Author Contributions

YH designed and supervised the experiments. PC, ZS, GL, and BW performed all the phenotypic evaluations. LM, YiL, PC, WD, and BP performed analysis and interpretation of the data. PC wrote the paper. GW, YaL, and DZ provided rice germplasm samples. GG, QZ, JX, and XL participated in the field management and logistic work.

## Conflict of Interest Statement

The authors declare that the research was conducted in the absence of any commercial or financial relationships that could be construed as a potential conflict of interest.

## Footnotes

1 http://ricevarmap.ncpgr.cn

2 https://tassel.bitbucket.io/

3 https://www.r-project.org/

4 http://crep.ncpgr.cn/crep-cgi/home.pl
