# Supplementary material for: Genetic Basis of Variation in Rice Seed Storage Protein (Albumin, Globulin, Prolamin, and Glutelin) Content Revealed by Genome-Wide Association Analysis
Source: Front Plant Sci. 2018 May 9;9:612. doi: 10.3389/fpls.2018.00612 (PMC5954490; doi:10.3389/fpls.2018.00612)
Supplement: Supplementary file 6 [file Data_Sheet_1.doc]

Title:

**Genetic basis of variation in rice seed storage protein (albumin, globulin, prolamin and glutelin) content revealed by genome-wide association analysis**

Authors:

**Pingli Chen^1^, Zhikang Shen^1^, Luchang Ming^1^, Yibo Li^1^, Wenhan Dan^1^, Guangming Lou^1^, Bo Peng^1^, Bian Wu^1^, Yanhua Li^2^, Da Zhao^1^, Guanjun Gao^1^, Qinglu Zhang^1^, Jinghua Xiao^1^, Xianghua Li^1^, Gongwei Wang^1^, Yuqing He^1^***

^1^National Key Laboratory of Crop Genetic Improvement and National Center of Plant Gene Research (Wuhan), Huazhong Agricultural University, Wuhan 430070, China

^2^Life Science and Technology Center, China National Seed Group Co., Ltd, Wuhan 430075, China

*Correspondence: Yuqing He (yqhe@mail.hzau.edu.cn**)**

Phone: +86-27-87281689

Fax: 86-27-87287092

Running title: GWAS of rice storage protein


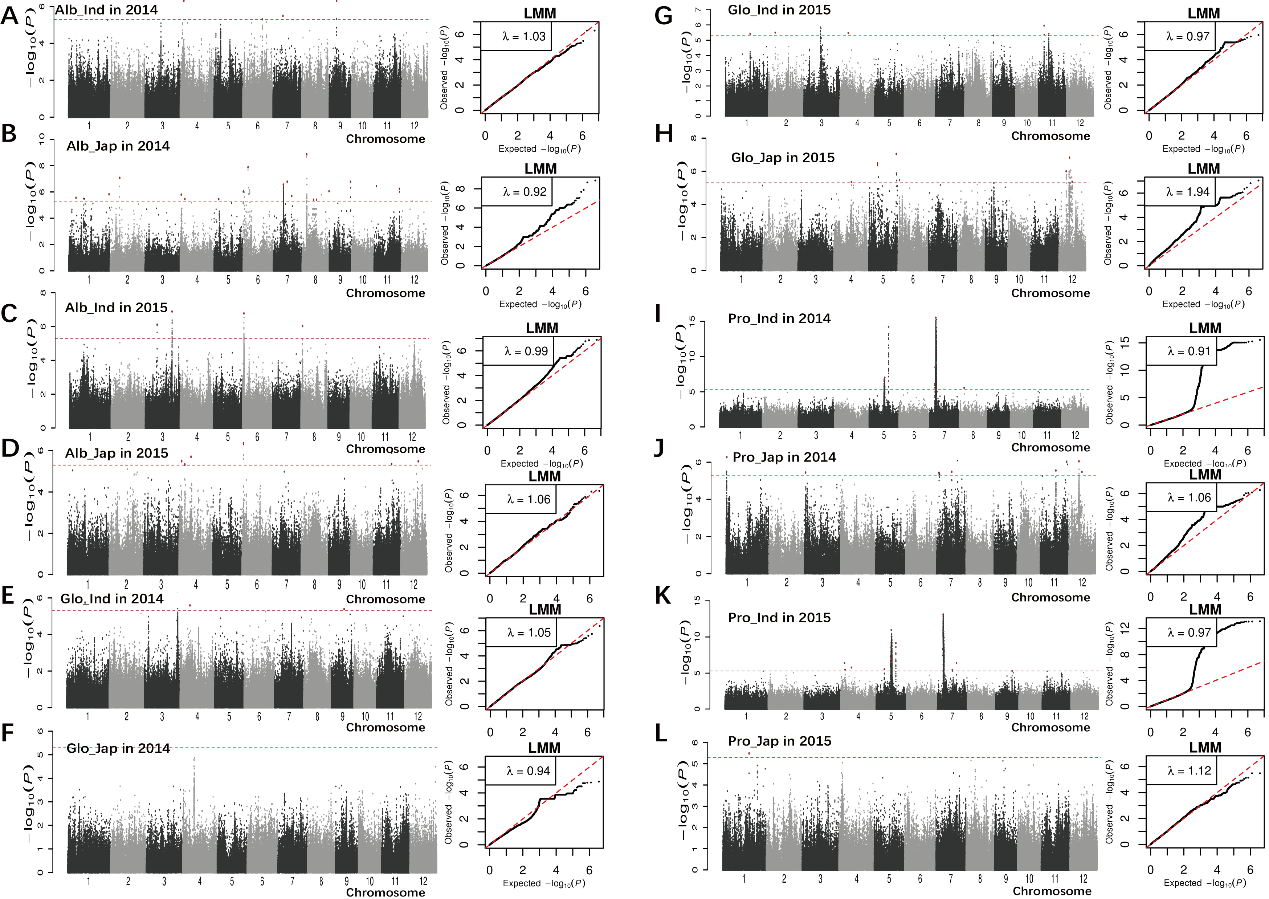


**Supplementary Figure S1** **Genome-wide *P*-values and quantile-quantile plots from LMM model for Alb (A-D), Glo (E-H) and Pro (I-L) in two years in the *indica* and *japonica* subpopulations are shown in 12 panels.**

The *x* axis depicts the physical location of SNPs across the 12 rice chromosomes and the *y* axis shows the -log10 (*P* value). Lead SNPs of significant peaks in red. The horizontal dotted line indicates the genome-wide significance threshold (*P* = 5.0E-06). *Ind*, *indica*; *Jap*, *japonica*.


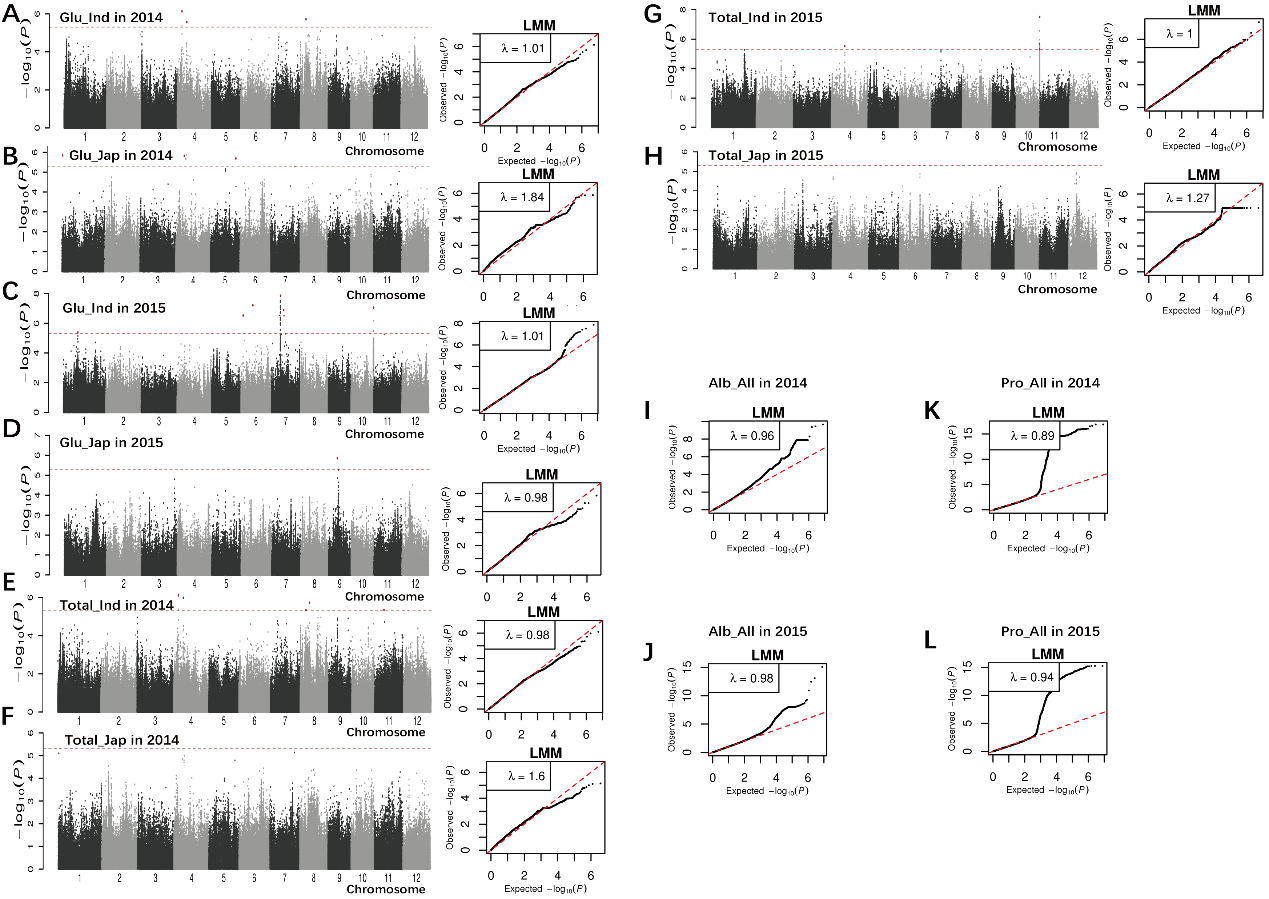


**Supplementary Figure S2** **Genome-wide *P*-values and quantile-quantile plots from LMM model for Glu (A-D), Total (E-H) in two years in the *indica* and *japonica* subpopulations, and quantile-quantile plots from LMM model for Alb (I-J) and Pro (K-L) in two years in the all population** **are shown.**

The *x* axis depicts the physical location of SNPs across the 12 rice chromosomes and the *y* axis shows the -log10 (*P* value). Lead SNPs of significant peaks in red. The horizontal dotted line indicates the genome-wide significance threshold (*P* = 5.0E-06). *Ind*, *indica*; *Jap*, *japonica*.

**Supplementary Table S1 Names, origin, population structure and grain storage protein contents of 527 *O. sativa* accessions.**

This table is in a separate sheet.

**Supplementary Table S2 Primer pairs for qRT-PCR.**

This table is in a separate sheet.

**Supplementary Table S3 Descriptive statistics of five traits in the different subpopulations for two years.**

| **Trait** | **Year** |  | ***Ind*** | | |  |  | ***Jap*** | | |  | ***H*^2^_B_^d^ (%)** |
| --- | --- | --- | --- | --- | --- | --- | --- | --- | --- | --- | --- | --- |
|  |  | **N**^a^ | **Mean ± SD**^b^ **(mg/g)** | **Range**  **(mg/g)** | **CV**^c^ **(%)** |  | **N** | **Mean ± SD (mg/g)** | **Range**  **(mg/g)** | **CV (%)** |  |  |
| Alb | 2014 | 244 | 2.8 ± 0.7 | 0.9 - 5.3 | 25.0% |  | 98 | 3.3 ± 1.2 | 1.6 - 6.9 | 36.4% |  | 63.4 |
|  | 2015 | 276 | 3.2 ± 0.8 | 0.5 - 5.6 | 25.0% |  | 139 | 3.6 ± 1.0 | 0.5 - 6.5 | 27.8% |  |  |
| Glo | 2014 | 238 | 5.8 ± 0.9 | 2.1 - 8.2 | 15.5% |  | 95 | 5.9 ± 1.0 | 3.2 - 8.1 | 16.9% |  | 49.4 |
|  | 2015 | 288 | 5.2 ± 1.0 | 1.5 - 8.1 | 19.2% |  | 153 | 5.7 ± 1.2 | 1.7 - 10.5 | 21.1% |  |  |
| Pro | 2014 | 244 | 2.7 ± 1.6 | 0.1 - 7.9 | 59.3% |  | 98 | 2.7 ± 1.0 | 0.5 - 6.3 | 37.0% |  | 76.8 |
|  | 2015 | 285 | 2.1 ± 1.4 | 0.1 - 6.8 | 66.7% |  | 149 | 2.0 ± 0.9 | 0.1 - 4.7 | 45.0% |  |  |
| Glu | 2014 | 244 | 59.1 ± 10.8 | 34.4 - 90.7 | 18.3% |  | 98 | 59.3 ± 11.2 | 38.4 - 93.3 | 18.9% |  | 30.3 |
|  | 2015 | 285 | 42.8 ± 7.6 | 25.2 - 65.5 | 17.8% |  | 151 | 45.5 ± 8.4 | 23.3 - 67.8 | 18.5% |  |  |
| Total | 2014 | 238 | 70.5 ± 11.9 | 44.1 - 103.6 | 16.9% |  | 95 | 71.4 ± 12.3 | 46.9 - 106.7 | 17.2% |  | 29.5 |
|  | 2015 | 257 | 52.7 ± 8.2 | 32.6 - 80.1 | 15.6% |  | 130 | 56.3 ± 8.8 | 33.5 - 78.4 | 15.6% |  |  |

^a^N, number of accessions; ^b^SD, standard deviation; ^c^CV, coefficient of variation; ^d^*H*^2^_B_, broad sense heritability. Total, total SSP content; *Ind*, *indica*; *Jap*, *japonica*.

**Supplementary Table S4 Pearson’s correlation coefficients among** **SSPs** in two environments.

|  | **Alb** | **Glo** | **Pro** | **Glu** | **Total** |
| --- | --- | --- | --- | --- | --- |
| Alb |  | 0.25^***^ | 0.10^*^ | 0.34^***^ | 0.42^***^ |
| Glo | 0.06 |  | 0.09 | 0.37^***^ | 0.45^***^ |
| Pro | -0.05 | 0.01 |  | 0.22^***^ | 0.34^***^ |
| Glu | 0.12^**^ | 0.25^***^ | 0.24^***^ |  | 0.99^***^ |
| Total | 0.20^***^ | 0.35^***^ | 0.39^***^ | 0.98^***^ |  |

Upper diagonal, correlation coefficients in 2014; bottom diagonal, correlation coefficients in 2015. **P*<0.05, ***P*<0.01 and ****P*<0.001.

**Supplementary Table S5 Associated SNPs identified by the LMM method in the *indica* and *japonica*.**

This table is in a separate sheet.

**Supplementary Table S6** **Haplotype analysis of seven genes *RP6*, *RM1*, *Wx*, LOC_Os05g26240, LOC_Os05g25500, LOC_Os03g29750 and, LOC_Os02g13130.**

Haplotypes in all accessions (haplotypes with less than five accessions were omitted) according to SNP data from RiceVarMap based on MSU6.1 annotation. The region contained coding region and 2 kb upstream of the gene. The number of accessions and trait value in each haplotype are shown in the right columns. Hap., haplotype.

This table is in a separate sheet.

**Supplementary Table S7 Relative expression of *RP6*, *RM1*, and *Wx* in accessions randomly chosen from *indica* or *japonica* subpopulation with different haplotypes.**

This table is in a separate sheet.

**Supplementary Table S8 Association analyses of known genes based on haplotypes identified in two environments.**

| **Gene** | **N^a^** | **Trait** | **Population** | ***P* (2014)** | ***P* (2015)** |
| --- | --- | --- | --- | --- | --- |
| *AGPS2a* | 6 | Alb | All | 9.3E-10 | 2.9E-11 |
| *GBSSII* | 10 | Glu | All |  | 5.7E-04 |
| *OsACS6* | 5 | Glo | All |  | 3.9E-07 |
| *GluA1* | 6 | Total | All |  | 2.6E-10 |
| *ISA2* | 5 | Alb | All | 2.4E-07 | 4.3E-13 |
| *OsAAT2* | 7 | Alb | All | 1.1E-06 | 3.5E-13 |
| *OsAAT2* | 5 | Total | All |  | 1.0E-07 |
| *Wx* | 7 | Alb | All | 2.2E-09 | 1.2E-24 |
| *Wx* | 6 | Alb | *Ind* |  | 5.7E-07 |
| *Wx* | 2 | Alb | *Jap* |  | 1.2E-07 |
| *PUL* | 6 | Pro | All | 5.0E-06 | 4.2E-04 |
| *PUL* | 9 | Pro | *Ind* | 1.4E-04 |  |
| *RA17* | 3 | Pro | All | 5.6E-34 | 1.9E-17 |
| *RA17* | 2 | Pro | *Ind* | 3.0E-27 | 5.4E-19 |
| *RA16* | 4 | Pro | All | 1.1E-37 | 2.8E-25 |
| *RA16* | 3 | Pro | *Ind* | 2.0E-28 | 1.5E-18 |
| *RM1* | 3 | Pro | All | 7.0E-25 | 2.0E-34 |
| *RM1* | 2 | Pro | *Ind* | 3.9E-19 | 4.6E-28 |
| *RP6* | 3 | Pro | All | 7.5E-25 | 5.0E-27 |
| *RP6* | 2 | Pro | *Ind* | 6.5E-20 | 2.5E-29 |

^a^N, number of haplotypes in accessions; *P*, *P*-value in ANOVA. *Ind*, *indica*; *Jap*, *japonica*.
